# Comparison of Blood-Based Shotgun and Targeted Metagenomic Sequencing for Microbiological Diagnosis of Infective Endocarditis

**DOI:** 10.1093/ofid/ofad546

**Published:** 2023-10-31

**Authors:** Laure Flurin, Cody R Fisher, Matthew J Wolf, Bobbi S Pritt, Daniel C DeSimone, Robin Patel

**Affiliations:** Divisions of Clinical Microbiology, Mayo Clinic, Rochester, Minnesota, USA; Department of Intensive Care, University Hospital of Guadeloupe, Pointe-à-Pitre, France; Division of Infectious Diseases, Mayo Clinic, Rochester, Minnesota, USA; Divisions of Clinical Microbiology, Mayo Clinic, Rochester, Minnesota, USA; Division of Infectious Diseases, Mayo Clinic, Rochester, Minnesota, USA; Divisions of Clinical Microbiology, Mayo Clinic, Rochester, Minnesota, USA; Division of Infectious Diseases, Mayo Clinic, Rochester, Minnesota, USA; Division of Infectious Diseases, Mayo Clinic, Rochester, Minnesota, USA; Division of Infectious Diseases, Mayo Clinic, Rochester, Minnesota, USA; Divisions of Clinical Microbiology, Mayo Clinic, Rochester, Minnesota, USA; Division of Infectious Diseases, Mayo Clinic, Rochester, Minnesota, USA

**Keywords:** infective endocarditis, next-generation sequencing, shotgun metagenomics, targeted metagenomics, the Karius test

## Abstract

**Background:**

Shotgun and targeted metagenomic sequencing have been shown in separate studies to be potentially useful for culture-free pathogen identification in blood and/or plasma of patients with infective endocarditis (IE). However, the 2 approaches have not been directly compared. The aim of this study was to compare shotgun metagenomic sequencing with targeted metagenomic sequencing (tMGS) for organism identification in blood or plasma of patients with IE.

**Methods:**

Patients with possible or definite IE were prospectively enrolled from October 2020 to July 2021. Shotgun metagenomic sequencing was performed with the Karius test, which uses microbial cell-free DNA (mcfDNA) sequencing to detect, identify, and quantitate DNA-based pathogens in plasma. tMGS was performed using a 16S ribosomal RNA (rRNA) polymerase chain reaction assay targeting the V1 to V3 regions of the 16S rRNA gene. Results were compared using the McNemar test of paired proportions.

**Results:**

Samples from 34 patients were investigated. The Karius test was positive in 24/34 (71%), including 3/6 (50%) with blood culture–negative endocarditis (BCNE), which was not significantly different from the positivity rate of tMGS (*P* = .41). Results of the Karius test were concordant with tMGS in 75% of cases. The Karius test detected 2 cases of methicillin-resistant *Staphylococcus aureus* among the 7 *S. aureus* detections, in accordance with results of phenotypic susceptibility testing. The combination of blood cultures, the Karius test, and tMGS found a potential causative pathogen in 33/34 (97%), including 5/6 with BCNE.

**Conclusions:**

The Karius test and tMGS yielded comparable detection rates; however, beyond organism identification, the Karius test generated potentially useful antibiotic resistance data.

## Teaser

The Karius test (sMGS) and tMGS had similar positivity rate in blood samples from 34 patients with IE. Overall, positivity of both tests and blood cultures combined was 97%. Either method has potential in routine practice for culture-free diagnostic.

In the United States, infective endocarditis (IE) affects 3–10 per 100 000 people per year, with a mortality rate of 15% [1, 2]. Success of antibiotic treatment for this devastating disease is highly dependent on identification of the pathogen(s) responsible for the infection. Delayed diagnosis and initiation of adequate antibiotic therapy increase morbidity and mortality [3]. Conventional laboratory tools for initial microbiologic diagnosis of IE are limited to blood cultures and serology for *Bartonella* species and *Coxiella burnetii­*. These tools are often helpful in diagnosing blood culture–positive endocarditis (BCPE). However, blood culture–negative endocarditis (BCNE) accounts for 40%–71% of cases, most of which are not caused by *Bartonella* species or *C. burnetii* [4, 5]. Instead, most BCNE cases are due to antecedent antibiotic treatment, with slow-growing or noncultivable microbes making up ∼10% of BCNE [6, 7].

Limitations of culture-based methods may be circumvented by molecular-based methods for BCNE. Nucleic acid amplification tests (NAATs), including 16S ribosomal RNA (rRNA) gene polymerase chain reaction (PCR) assays followed by Sanger sequencing on valve tissues, have improved detection of organisms in BCNE compared with valve cultures [8–11]. On the other hand, 16S rRNA gene PCR followed by Sanger sequencing had poor sensitivities on both blood and plasma due to low concentrations of bacterial DNA in these samples [12]. Low specimen volume inputs such as <1 mL may decrease sensitivity compared with larger volume blood cultures. An estimated 40%–50% of patients with IE do not undergo valve replacement surgery [13, 14]. Accordingly, there is a need for fast and accurate pathogen identification in patients with suspected or proven IE using a noninvasive, easy-to-collect sample, such as blood (or plasma).

Newer methods, such as next-generation sequencing, have deeper sequencing capabilities compared with Sanger sequencing, with the potential to identify pathogens where traditional blood cultures and Sanger sequencing approaches have failed [15]. Next-generation sequencing–based approaches may detect difficult-to-grow pathogens, including those that are nonculturable because of previous antibiotic therapy, and may even detect resistance genes. In 2019, Blauwkamp et al. described the validation and implementation of a shotgun metagenomic sequencing (sMGS) assay called the Karius test, which detects microbial cell-free DNA (mcfDNA) in plasma [16]. In 2021, a potential use of the Karius test was described in a retrospective case series of 10 pediatric patients with IE, in which 8 out of 10 pathogens were identified [17]. Another study assessed the sensitivity of the Karius test in 23 adult patients with definite IE, mainly caused by *Staphylococcus aureus*; the sensitivity was 87% for both blood cultures and the Karius test, although the sensitivity in BCNE was only 33% for the Karius test [18]. In the first prospective study of patients with IE, we evaluated 16S rRNA-based targeted metagenomic sequencing (tMGS) blood- and plasma-based assays in 34 patients with possible and definite endocarditis; the positivity rate of the tMGS assay was 66% overall, and 83% in BCNE, independent of previous antibiotic treatment [19].

Compared with tMGS, the Karius test has the advantage of being an exhaustive test, able to detect bacteria, viruses (DNA only), fungi, parasites, and some resistance genes, but with the limitation of being costly and more labor-intensive [15]. In comparison, 16S ribosomal RNA gene-based tMGS may be more rapid, easier to perform, and equally sensitive for detection of bacteria. A previous study comparing sMGS and tMGS of sonicate fluid for diagnosis of periprosthetic joint infection found no difference in positive and negative agreement between the 2 approaches [20]. Until now, tMGS and sMGS approaches have not been directly compared for diagnosis of IE in blood. The aim of this study was to compare the Karius test with tMGS for organismal identification in blood or plasma in a prospective cohort of patients with IE.

## METHODS

### Study Design

Subjects with a suspicion of IE were screened and approached for consent from October 2020 to July 2021 as previously described [19]. Only possible or definite IE cases were included, according to the modified Duke criteria [5]. Minors (<18 years), pregnant women, inmates, subjects who refused to consent, and subjects who were discharged or died before blood draw were excluded. Subjects were prospectively enrolled at an early phase of IE diagnosis, but no time cutoff was applied for inclusion. A single 20-mL blood draw was collected and divided into two 10-mL EDTA Vacutainer tubes (BD, Franklin Lakes, NJ, USA). One EDTA tube was aliquoted into 10 cryovials of 1 mL of whole blood, and the other centrifuged and residual plasma was aliquoted into 5 cryovials of 1 mL each. All cryovials were immediately frozen and stored at −80°C.

### Shotgun Metagenomic Sequencing—Karius Test

For the Karius test, which detects mcfDNA in plasma, frozen plasma was shipped to Karius for mcfDNA sequencing, which was performed by sequential processes of automated DNA extraction, conversion to DNA libraries, and sequencing using Illumina NextSeq 500. Controls for carryover, bias, sequencing quality, mix-ups, and quantitation were included. Sequencing results were run through the Karius bioinformatics pipeline (version 3.11) to reduce human cross-reactivity calls and align microbial sequences to a curated database of microbe assemblies. Microbes with a signal significantly higher than that detected in >680 healthy donors were reported qualitatively and quantitated as molecules per μL, which quantifies each organism identified by representing the number of DNA sequencing reads present per μL of plasma [16]. For samples in which *S. aureus* was detected, the Karius bioinformatics pipeline identified mcfDNA fragments arising from SCC*mec*, a genetic element associated with methicillin resistance. The pipeline reported methicillin-resistant or -susceptible *S. aureus* based on the quantity of SCC*mec* fragments found. False positives were controlled by analysis of sequence similarity to non-*mec* SCC and the presence of coagulase-negative staphylococci in the sample.

### Targeted Metagenomics Sequencing

Plasma and whole-blood samples had previously undergone DNA extraction and 16S rRNA gene amplification followed by next-generation sequencing, as reported [19]. A positive tMGS result was determined by a positive plasma and/or whole-blood tMGS test.

### Statistical Analysis

Qualitative values were compared using the *t* test or Fisher exact test, as appropriate. Quantitative values were tested for normality using the Kolmogorov-Smirnov test and compared using Mann-Whitney or unpaired *t* tests, as appropriate. A log-rank (Mantel Cox) test was used to compare the cumulative percentage of positive tests. To compare positivity of tests (percentages), McNemar's test of paired proportions was performed. *P* values <.05 were considered statistically significant.

### Patient Consent

Written consent was obtained from the patient, or his/her legal representative if not able to consent himself/herself. The design of the work was approved by the local ethical committee, the Mayo Clinic Institutional Review Board (18–006497).

## RESULTS

### Patients’ Characteristics

**Figure 1.**
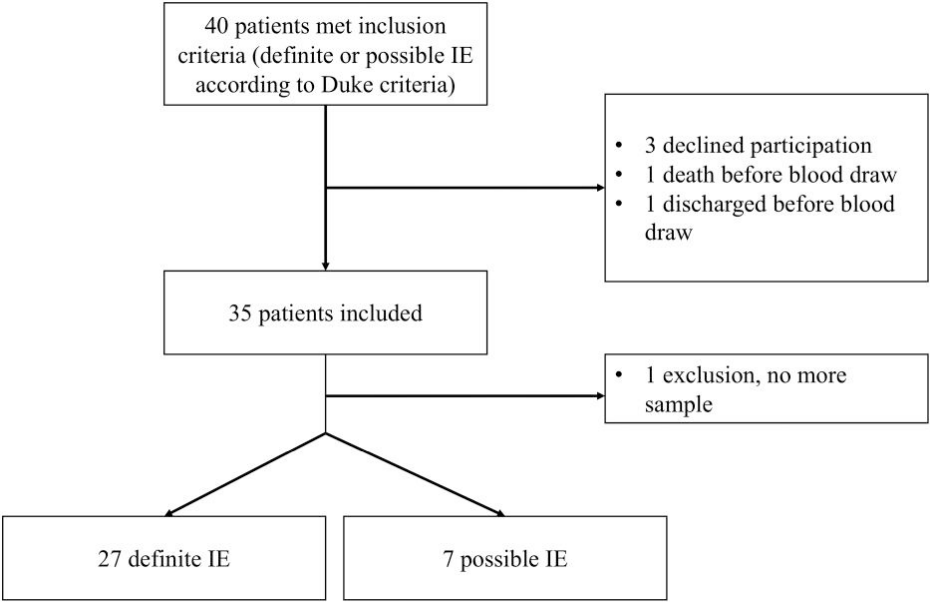
Flowchart. Abbreviation: IE, infective endocarditis.

Of the 40 subjects screened, 34 were included, 27 with definite IE and 7 with possible IE (Figure 1). Subjects were mainly males with a median age of 70 years. IE predominantly involved the aortic valve (68%) and a prosthetic valve (65%) which was either mechanical (32.5%) or biological (32.5%). Nine subjects (26%) had a history of previous IE. Six subjects had blood culture–negative endocarditis (BCNE) (Table 1).

**Table 1.** Patient Characteristics

Patient Characteristics	Total (n = 34)
Age, y	70 [56–82]
Sex, female	7 (21)
BMI, kg/m^2^	29 [23–34]
Hypertension	26 (76)
Diabetes, type 2	13 (38)
Chronic heart failure	19 (56)
Immunosuppressed	4 (12)
Injection drug use	2 (6)
Aortic	23 (68)
Mitral	7 (21)
Pulmonary	1 (3)
Tricuspid	5 (15)
Prosthetic valve	22 (65)
Bioprosthesis	11 (32.5)
Mechanical prosthesis	11 (32.5)
Multiple valves infected	3 (9)
Previous endocarditis	9 (26)
Duke criteria for endocarditis	
Definite	27 (79)
Possible	7 (21)
Blood culture–negative endocarditis	6 (18)
Laboratory findings, at inclusion	
Hemoglobin, mg/dL	9.5 [8.6–12]
Total leukocyte count, 10^9^/L	9.1 [7.4–14]
Neutrophil count, 10^9^/L	7.2 [5.7–11]
Platelet count, 10^9^/L	198 [97–255]
C-reactive protein (n = 28/40), mg/L	100 [51–192]
Erythrocyte sedimentation rate, mm/h	54 [33–82]
Ultrasound	
TTE, positive for IE/total of TTE	11/29 (38)
TEE, positive for IE/total of TEE	31/34 (91)
Complications	
Acute heart failure	16 (47)
Perivalvular or septal/myocardial abscess	11 (32)
Peripheral septic emboli	16 (47)
Death	6 (17)
Treatment	
Valve replacement surgery	15 (44)
Positive valve culture	6/13 (46)
Pathologic lesions on valve tissue	13/13 (100)
Time since last positive culture and study test, d	5 [0.5–7]
Time since first antibiotic dose and study test, d	5 [3.5–9.5]

Data are presented as No. (%) or median [interquartile range].

Abbreviations: IE, infective endocarditis; TEE, Transthoracic Echocardiogram; TTE, Transesophageal Echocardiogram.

### Performance of the Karius Test in IE

**Figure 2.**
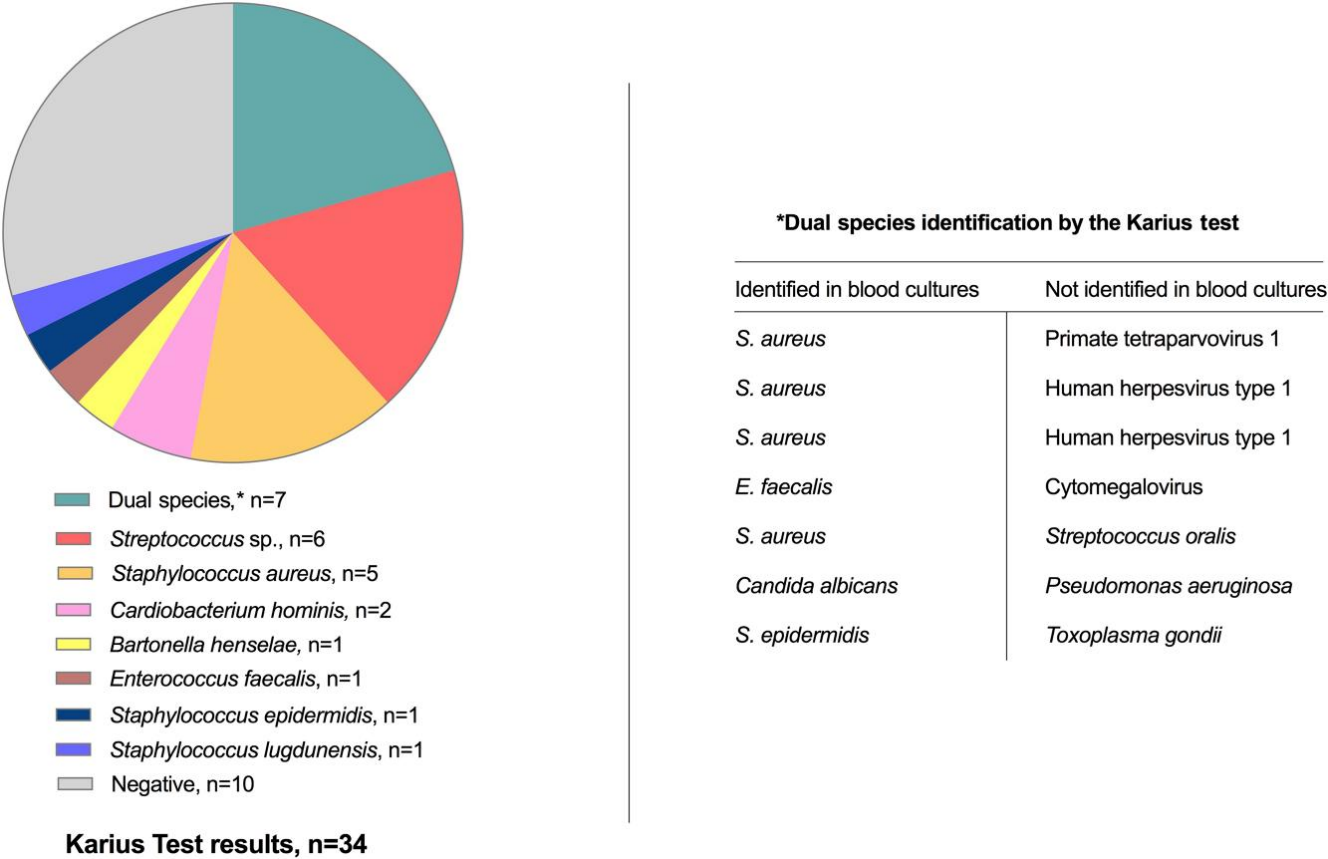
Microbiological identification by the Karius test.

The Karius test was positive in 24/34 (71%), including 3/6 (50%) with BCNE and 21/28 (75%) with BCPE (Table 2). Of 24 positive Karius test results, 30 organisms yielded positive results at a median molecules per milliliter value (interquartile range) of 2515 (550–16 740). *S. aureus* was identified in 9 samples (5 monomicrobial and 4 dual species), of which 2/9 (22%) had methicillin resistance genetically detected, in accordance with the results of phenotypic susceptibility testing. In the 17 samples with monomicrobial detections*, Streptococcus* species (1 *Streptococcus agalactiae*, 1 *Streptococcus dysgalactiae*, 1 *Streptococcus lutetiensis*, 2 *Streptococcus oralis*, and 1 *Streptococcus salivarius*) were identified in 6, followed by 5 *S. aureus*, 2 *Cardiobacterium hominis*, 1 *Bartonella henselae*, 1 *Enterococcus faecalis*, 1 *Staphylococcus epidermidis*, and 1 *Staphylococcus lugdunensis* (Figure 2).

**Table 2.** Comparison of Karius Test and tMGS Results

	Positive Blood Culture Result at Time of Study Blood Draw	Any Positive Blood Culture	Positive Karius Test	Positive tMGS
Total (n = 34)	7 (21)	28 (82)	24 (71)	22 (65)
BCPE (n = 28)	7 (25)	28 (100)	21 (75)	17 (61)
BCNE (n = 6)	0 (−)	0 (−)	3 (50)	5 (83)

Data are presented as No. (%).

Abbreviations: BCNE, blood-culture negative endocarditis; BCPE, blood-culture positive endocarditis; tMGS, targeted metagenomic sequencing.

The Karius test resulted in dual species detections in 7/34 samples (Figure 2). In 2 samples, the Karius test identified *S. aureus* with herpes simplex virus type 1, and in another, *S. aureus* with primate tetraparvovirus 1 of unknown clinical significance. In 1 sample, *E. faecalis* was detected with herpes simplex virus type 1. In 1 patient, the Karius test detected *Candida albicans* and *Pseudomonas aeruginosa*; *P. aeruginosa* was not found in either blood cultures or tMGS. In another patient with positive *S. aureus* cultures, the Karius test also detected *Streptococcus oralis*. Finally, 1 sample, from an immunocompromised patient with possible IE, yielded *S. epidermidis* and *Toxoplasma gondii*. The Karius test was negative in 10 subjects, 3 with BCNE and 7 with positive blood cultures, albeit not at the time of the study blood draw.

### Comparison of the Karius Test and tMGS

**Figure 3.**
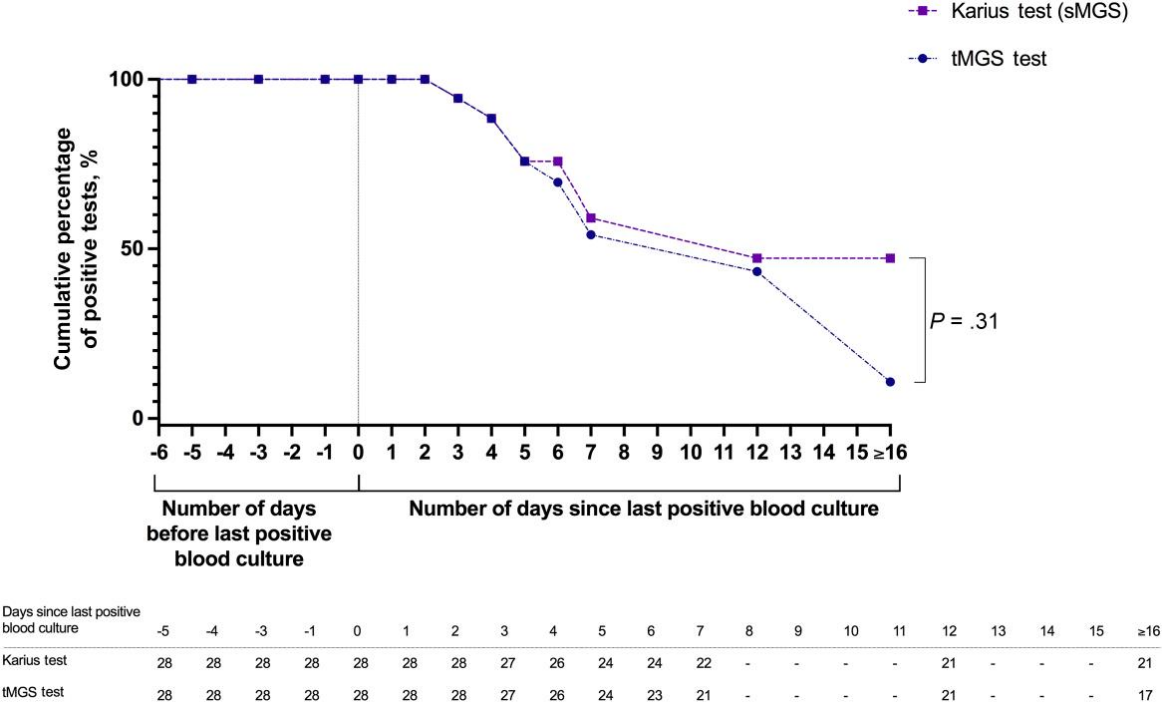
Cumulative positivity of the Karius test and tMGS test in BCPE by the number of days since the last positive culture. Abbreviations: BCPE, blood culture–positive endocarditis; sMGS, shotgun metagenomic sequencing; tMGS, targeted metagenomic sequencing.

tMGS was positive in 22/34 (65%), including 5/6 (83%) with BCNE. BCPE results were concordant with the Karius test in 75% (22/28) of cases. In BCPE, 100% (10/10) of tests were positive by both the Karius test and tMGS through 2 days from the last positive blood culture, and both tests detected the same organisms in these 10 samples. Beyond 2 days from the last positive blood culture, positivity was 61% (11/18) for the Karius test and 39% (7/18) for tMGS. Concordance of positive detections by tMGS and the Karius test was 100% (16/16) through 5 days from the last positive blood culture. Beyond 5 days of the last positive culture, concordance was only 50% (6/12) between the 2 tests (Table 3, Figure 3).

**Table 3.** Comparison of the Karius Test, tMGS, and Blood Cultures

Last Positive Blood Culture, d	Blood Culture Result at Time of Study Blood Draw	Karius Test	tMGS	Any Positive Blood Culture
**Blood Culture–Positive Endocarditis**				
−5	*Staphylococcus aureus* (MR)	*S. aureus* (MR)	*S. aureus* complex	*S. aureus* (MR)
−3	*S. aureus* (MR)	Primate tetraparvovirus 1, *S. aureus* (MR)	*S. aureus* complex, *Streptococcus mitis* group	*S. aureus* (MR)
−3	*Enterococcus faecalis*	Cytomegalovirus*, E. faecalis*	*E. faecalis*	*E. faecalis*
−1	*S. aureus*	*S. aureus*	*S. aureus* complex	*S. aureus*
−1	*S. aureus*	*S. aureus,* Human herpesvirus type 1	*S. aureus* complex	*S. aureus*
0	*Staphylococcus epidermidis*	*S. epidermidis*	*S. epidermidis*	*S. epidermidis*
0	*Staphylococcus lugdunensis*	*S. lugdunensis*	*S. lugdunensis*	*S. lugdunensis*
1	Negative	*S. aureus*	*S. aureus* complex	*S. aureus*
2	Negative	Human herpesvirus type 1, *S. aureus*	*S. aureus* complex	*S. aureus*
2	Negative	*Streptococcus oralis*	*Streptococcus mitis* group	*S. mitis* group
3	Negative	Negative	Negative	*E. faecalis*
3	Negative	*S. oralis*	*S. mitis* group	*S. mitis* group
4	Negative	Negative	Negative	*S. anginosus*
4	Negative	*E. faecalis*	*E. faecalis*	*E. faecalis*
5	Negative	Negative	Negative	*S. mitis* gp.
5	Negative	Negative	Negative	*S. aureus*
6	Negative	*Streptococcus salivarius*	*S. salivarius* group	*S. salivarius* group
6	Negative	*Staphylococcus epidermidis*, *Toxoplasma gondii*	*Staphylococcus hominis*	*Streptococcus anginosus*, *S. epidermidis*
6	Negative	*S. aureus*	Negative	*S. aureus*
7	Negative	Negative	*Corynebacterium amycolatum*	*C. amycolatum*
7	Negative	*Streptococcus agalactiae*	Negative	*S. agalactiae*
7	Negative	*Streptococcus lutetiensis*	*Streptococcus bovis* group	*S. lutetiensis*
7	Negative	Negative	Negative	*Enterococcus faecium*
12	Negative	Negative	Negative	*S. aureus*
16	Negative	*Candida albicans*, *Pseudomonas aeruginosa*	Negative	*C. albicans*
16	Negative	*S. aureus*, *S. oralis*	Negative	*S. aureus*
17	Negative	*Cardiobacterium hominis*	Negative	*C. hominis*
31	Negative	*S. aureus*	*S. aureus* complex	*S. aureus*
**Blood Culture–Negative Endocarditis**				
NA	Negative	*Bartonella henselae*	*B. henselae*	Negative
NA	Negative	*Streptococcus dysgalactiae*	*Streptococcus salivarius/thermophilus*	Negative
NA	Negative	*C. hominis*	*Kingella* species	Negative
NA	Negative	Negative	*Cutibacterium acnes*	Negative
NA	Negative	Negative	*Corynebacterium* species	Negative
NA	Negative	Negative	Negative	Negative

Subjects in whom the Karius test was positive, tMGS was negative, or vice versa are highlighted in blue.

Abbreviations: MR, methicillin-resistant; NA, not applicable; tMGS, targeted metagenomic sequencing.

In 3 subjects with BCNE, the Karius test and tMGS detected *B. henselae* and *Streptococcus* species. In 1 subject, the Karius test detected *C. hominis*, whereas tMGS detected *Kingella* species. This patient had completed 6 weeks of antibiotic treatment for *C. hominis* aortic mechanical IE and had returned 2 months post–treatment completion with evidence of definite IE relapse, including fever, new aortic vegetations, splenic emboli, and renal insufficiency, but with negative blood cultures. No valve replacement surgery was performed.

tMGS was positive in 2 additional patients with BCNE with negative Karius test results; in 1, tMGS detected *Cutibacterium acnes* in a patient with native tricuspid BCNE, and in another *Corynebacterium* species was detected in a subject with bioprosthetic aortic valve BCNE. To note, *Cutibacterium acnes* is not included in the Karius reportable database.

### Correlation to Last Positive Blood Culture

The positivity of both the Karius test and tMGS was 100% through 2 days after the last positive blood culture (Figure 3). Five days after the last positive blood culture, the positivity of both tests declined without a significant difference between the 2 (*P* = .32). The Karius test identified the same microorganisms in 9/12 subjects in whom blood cultures were positive 5 days before the study blood draw compared with 5/12 for tMGS. Both tests yielded positive results up to 31 days after the last positive culture.

### Correlation to Antibiotic Therapy

**Figure 4.**
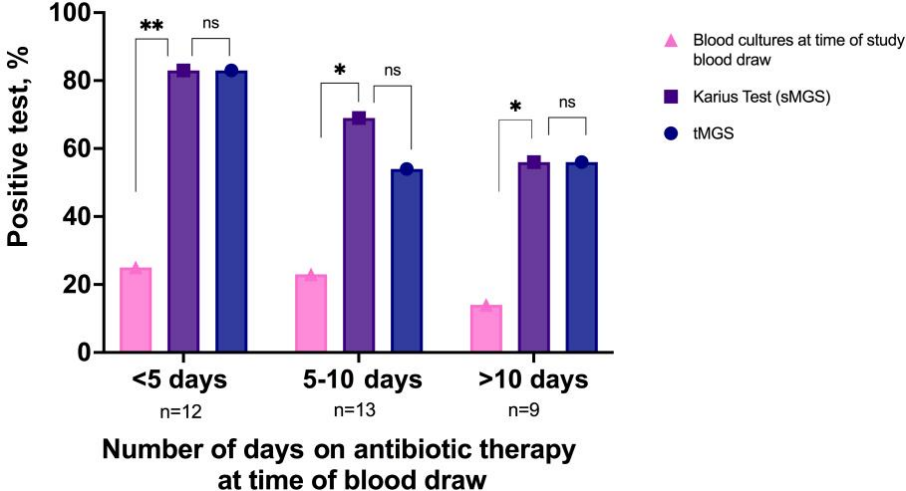
Positivity of the Karius test, tMGS, and blood cultures by the number of days on antibiotic therapy. *P < .05; **P < .01. Abbreviations: ns, not significant; sMGS, shotgun metagenomic sequencing; tMGS, targeted metagenomic sequencing.

All subjects had received at least 1 day of antibiotic treatment at the time of study blood draw (Figure 4). When the blood draw was completed from 1–4 days of antibiotic therapy, the positivity of blood cultures, tMGS, and the Karius test was 25%, 83%, and 83%, respectively, before 5 days of antibiotic treatment; 23%, 54%, and 65%, respectively, from 5 to 10 days of antibiotic treatment; and 14%, 56%, and 56%, respectively, after 10 days of antibiotic treatment.

The positivity of tMGS and the Karius test did not differ before 5 days (*P* = 1.0), from 5 to 10 days (*P* = .1), or over 10 days (*P* = 1.0) of antibiotic therapy. The positivity of blood cultures when collected at the same time as the study blood draw was significantly lower than the Karius test (sMGS) if the study blood draw was collected before the fifth day of antibiotic therapy (*P* = .008), from 5 to 10 days after the beginning of antibiotic therapy (*P* = .04), or after 10 days of antibiotic therapy (*P = .*04) (Figure 4).

The only subject with positive blood cultures and negative tMGS and Karius test results was a patient who had received suppressive antibiotics for >745 days for a previous *S. aureus* IE who had episodes of recurrent bacteriemia. At time of inclusion, the patient was diagnosed with relapse of IE involving the aortic and tricuspid valves complicated by aortic root abscess. The subject died of uncontrolled sepsis and cardiac failure, despite broad-spectrum antibiotic therapy, surgical valve replacement, and intensive care.

When combining results of blood cultures, the Karius test, and tMGS, an organism was detected in 97% of cases.

## DISCUSSION

To date, this is the first study to compare sMGS (the Karius test) with tMGS using blood-based assays in IE. Overall, the Karius test detected potential pathogens in 71% of cases, regardless of timing of blood collection, and 100% of BCPE cases if samples were collected within 2 days of the last positive blood culture. After this time, positivity for BCPE decreased to 61%. In a previous study, the Karius test had a reported sensitivity of 87% in BCPE [18]. The Karius test was positive in 3 of 6 subjects with BCNE.

The most common organisms identified by the Karius test were unsurprisingly streptococci and *S. aureus* [2]. An advantage of the Karius test compared with tMGS is its ability to identify nonbacterial organisms. *C. albicans* was found in a plasma sample that tMGS had missed and was found in blood cultures. The Karius test detected 4 viruses (2 herpes simplex virus type 1, 1 cytomegalovirus, 1 primate tetraparvovirus), all in BCPE; these viruses are unlikely to have been causes of IE. *T. gondii* was detected in addition to *S. epidermidis* in an immunosuppressed patient with sarcoidosis, with blood cultures positive for *S. epidermidis* and *S. anginosus*, and tMGS positive for *Staphylococcus hominis*. The clinical relevance of *T. gondii* detection in this case is unknown. In a study by Hogan et al., clinical impact analyses of the Karius test revealed excess antimicrobial treatment in 4% of cases because of unknown clinical relevance [21].

The Karius test correctly identified resistance genes in *S. aureus*–positive samples, in accordance with clinical testing. Although this is a small sample size, it is a promising tool for rapid bacterial resistance identification directly from blood, without the need for culture, as is required for current rapid detection methods [22].

Concordance in positivity between tMGS and the Karius test was 75%. The combination of tMGS, the Karius test, and blood culture resulted in a 97% detection rate in patients with IE. There were no significant differences in detection rates between the Karius test and tMGS overall (*P* = .47), in BCPE (*P* = .10), or in BCNE (*P* = .16). However, small albeit non–statistically significant differences between the Karius test and tMGS were noted in BCNE, with 60% and 83% detection rates (*P* = .16). A potential explanation could be the presence of intracellular bacterial DNA or debris within macrophages or neutrophils in whole blood, which could be missing in plasma [19, 23]. Also, tMGS is not impacted by human DNA background.

Discordance in positivity between tMGS and the Karius test was noted in 24% of cases, only in samples from subjects with BCNE and those with BCPE who had had negative blood cultures for >6 days. Concordance in positivity between tMGS and the Karius test was 100% through 5 days after the last positive blood culture. We hypothesize that after 5 days of negative blood cultures, circulating bacterial DNA concentrations are lower, and thus, the probability of pathogen detection lower. Moreover, as time on antibiotics elapses, the discordance in positivity and lower diagnostic yield increases over time. Indeed, the cumulative percentage of positive tests for both tMGS and the Karius test decreased with time from the first antibiotic dose and the last positive cultures. Eichenberger et al. proposed this phenomenon as a potential tool for monitoring endocarditis treatment and also reported that the Karius test could detect organisms through a median of 38.1 days after initiation of antibiotic therapy, based on repeated testing [18].

While no other study had evaluated differences between tMGS and sMGS in IE, a comparison in periprosthetic joint infection was recently published. Hong et al. investigated the diagnostic accuracy of sMGS vs tMGS in sonicate fluid and found similar positive percent agreements: 72% for tMGS and 73% (*P* = .83) for sMGS. Interestingly, another study found no significant difference between 16S rRNA PCR followed by Sanger sequencing and sMGS evaluated on 67 diverse tissue and fluid samples (including heart valves) [24]. However, all clinical metagenomics methods should be compared with caution. Results and sensitivities vary by sample type [25], DNA extraction method [26], amplification target used (for tMGS), library preparation, depth of sequencing, and bioinformatics platform [27–29].

Cost is an important consideration as the Karius test's listed price is $2000/sample. tMGS is estimated to cost 10 times less if performed in high volumes. Given that the Karius test does not deliver sequence data for local review/interpretation, it is not possible to apply a sequencing interpretation algorithm as described [19, 30] or have a clinical microbiology sequencing board re-interpret results [15]. In terms of turnaround time, tMGS can be delivered in 24 to 36 hours, and the Karius test in 26 hours from sample receipt to reportable results. However, these times do not include shipping times for send-out tests [31–33]. Conversely, tMGS does not detect nonbacterial organisms or resistance genes or quantify bacterial DNA (though the latter is technically possible), 2 advantages of the Karius test. This study is limited by its relatively small sample size and inclusion of patients regardless of the time since last positive culture.

In conclusion, the overall positivity of the Karius test was not different from detection of bacteria by tMGS in whole blood and plasma. The Karius test has the advantage of detection of fungal species and methicillin resistance in *S. aureus*. Whether using sMGS or tMGS, clinical metagenomics has the potential to provide culture-independent diagnoses in patients with IE.
